# Strengths, weaknesses, opportunities and threats of mobile applications in undergraduate nursing: a scoping review protocol

**DOI:** 10.1371/journal.pone.0314757

**Published:** 2025-04-03

**Authors:** Ana Clara Dantas, Leandro Melo de Carvalho, Bárbara Ebilizarda Coutinho Borges, Cyntia Leenara Bezerra da Silva, Ericles Lopes de Moura, João Pedro Machado de Lima, Allyne Fortes Vitor

**Affiliations:** Department of Nursing, Federal University of Rio Grande do Norte, Natal, Rio Grande do Norte, Brazil; Polytechnic Institute of Viana do Castelo, PORTUGAL

## Abstract

**Aim:**

To describe the protocol for a scoping review on the main strengths, weaknesses, opportunities and threats of mobile applications in undergraduate nursing.

**Background:**

Some studies have shown that mobile applications have been objects of increasing interest in nursing education. Although these technologies offer potential benefits to the educational process, their implementation and use are not always without challenges and implications. In this sense, the SWOT analysis will be used to summarize the strengths, weaknesses, opportunities and threats of those technologies.

**Design:**

Scoping review protocol, conducted in accordance with JBI guidelines.

**Method:**

This is a protocol for a scoping review to be carried out following the JBI guidelines, in three phases and in the following data sources: Nursing Database, Latin American and Caribbean Literature in Health Sciences, Medical Literature Analysis and Retrieval System Online, Scopus, Web of Science and ScienceDirect, later, Google Scholar and, finally, in the references of the primarily collected studies. The inclusion criteria are studies that addressed the topic of mobile applications in Nursing teaching, available in full, free of charge and without time or language cut-off. The exclusion criteria are studies that did not address the topic, opinion articles, letters to the editor and editorials. The results will be presented through tables and a SWOT chart, with the study variables.

**Expected Results:**

The results will cover the characterization of the sample studies, followed by a SWOT chart containing the main strengths, weaknesses, opportunities and threats of mobile applications in undergraduate nursing.

**Conclusion:**

The SWOT analysis will identify strengths and weaknesses, promote self-knowledge and strategies for mobile applications in undergraduate nursing. It can also assist in decision-making, communication with partners and publication, improving scientific research.

## Introduction

Technological advancement manifests itself through significant changes in the most varied sectors of our lives. Since the last century, society has witnessed the rapid expansion of computing, communications, biotechnology, and artificial intelligence. These advances have generated profound changes in the economy, education, and health, promoting greater global connectivity, productive efficiency, and innovative possibilities for the future [1,2].

Information and Communication Technologies (ICT) can be defined as a set of resources, systems and devices that allow the collection, storage, processing, transmission and sharing of information and data, as well as communication between people or machines. They can act in the modernization and development of society, affecting areas such as the economy, education, health, entertainment, among others. This includes computers and mobile devices [3].

Mobile applications, also known as apps, are software programs developed specifically to run on mobile devices, such as smartphones and tablets, and provide services and functionalities to users, making it easier to perform tasks, access information and communicate [4].

In the clinical educational setting, the use of smartphones to send text messages enhances immediate communication between preceptors, faculty, and students. Additionally, access to online resources such as videos, podcasts, practice guidelines, and pharmacological information assists in providing safe, evidence-based care [5].

With all the technological advances, today’s university professors face the challenge of engaging a generation of students with very different characteristics from previous generations. Generation Z, made up of those born in the 2000s and beyond, grew up in an environment of intense global connectivity, largely shaped by social networks. For these young people, constant access to information and continuous communication are natural elements of everyday life [6].

This generation stands out for its strong dependence on technology, using it as the main means to access information, interact with others, share ideas, create content and expand their knowledge [6]. Therefore, it is essential to reflect on the benefits and disadvantages to students in relation to the use of technology in teaching.

Research shows that around 90% of students use mobile applications as technological support to access educational information online [7]. Questions about regulatory policies and ethics, limited resources and the use of smartphones for non-educational purposes are challenges presented in the literature [8]. Thus, the development of the proposed study is justified by the importance of better understanding all aspects that permeate the use of mobile applications in nursing education.

A study on smartphone use by undergraduate nursing students indicates that smartphone applications promote active learning and long-term knowledge retention [9]. Another study showed that the use of interactive apps like Kahoot! has benefits in helping to maintain attention and facilitate feedback between teacher and student [10].

## Background

Some studies have shown that mobile applications have been objects of increasing interest in nursing education [11–12]. Although these technologies offer potential benefits to the educational process, their implementation and use are not always without challenges and implications [13,14].

In this sense, the SWOT analysis, often used in corporatism, will be used to summarize the potentialities and challenges of those technologies. In addition, the choice to develop a protocol for scoping review is aligned with the understanding that it is the most appropriate type of study in this case compared to other evidence synthesis approaches, since it aims to systematically map the literature on the topic presented in a broad way, with the aim of identifying the main key concepts, investigating the dimension and possible existing knowledge gaps [15].

### Aims

From this perspective, the following research question was raised: “What are the strengths, weaknesses, opportunities and threats of mobile applications in undergraduate nursing?”

## Methods

### Study

This is a scoping review protocol. A scoping review protocol holds significant importance as it establishes beforehand the scoping review’s objectives, methodologies, and reporting procedures, ensuring transparency throughout the process [16]. To ensure the quality of the study and its writing, this review will comply with the JBI guidelines and the PRISMA-ScR (Preferred Reporting Items for Systematic Reviews and Meta-Analyses extension for Scoping Reviews), according to S1 Checklist [16–17].

It is worth noting that the report of this protocol was guided by the best practice guidance and reporting items for the development of scoping review protocols [15].

### Registrations

Initially, a planning protocol was prepared and duly registered on an open science platform, the Open Science Framework, containing all the fundamental information for conducting the study. This protocol is available for access through the following electronic address https://osf.io/5j7cq/. This record details the research topic, the question of interest, the objective, the search strategy with the respective data sources, the descriptors used, the crossings carried out, as well as the criteria adopted for the selection of studies, in addition to the description of the instrument used for data extraction.

The study originated from the following research question: “What are the strengths, weaknesses, opportunities and threats of mobile applications in undergraduate nursing?” This question was schematized using the PCC mnemonic, as follows: the “P” represents the population (Undergraduate nursing students and teachers), the first “C” refers to the Concept, which are the Strengths, Weaknesses, Opportunities and Threats; and the second “C” refers to Context, which is Mobile applications, according to Table 1.

**Table 1:** Description and characterization of the PCC mnemonic used in the research.

Population/Problem: Undergraduate nursing students and teachers	Individuals enrolled in higher-level courses, which are offered by educational institutions, where students receive theoretical and practical training to become nursing professionals and teachers are responsible for the content taught and the development of professional practice.
**Concept:** Strengths, weaknesses, opportunities and threats	Strengths: represent positive and intangible internal attributes, which are under the control of the organization. Weaknesses: these are negative attributes that can hinder the ability to achieve established objectives. Opportunities: refer to attractive external factors that can be used to benefit the organization. Threats: are external and uncontrollable attributes that represent potential risks to the organization’s purpose.[18]
**Context:** Mobile applications	Software programs designed specifically to run on mobile devices, such as smartphones and tablets, that provide services and functionality to users, making it easier to perform tasks, access information and communicate.(4)

### Inclusion criteria

The inclusion criteria will be studies that address the topic of mobile applications used in Nursing undergraduate courses, available in full, free of charge and without time or language. Experimental, observational studies and reviews will be included. The exclusion criteria will be studies that do not address the topic, opinion articles, experience articles, letters to the editor, editorials, comments, abstracts, and protocols.

### Information sources

The study will follow literature guidelines, with a search strategy carried out in three distinct phases. In the first phase, six data sources will be consulted, namely: Nursing Database, Latin American and Caribbean Literature in Health Sciences, Medical Literature Analysis and Retrieval System Online, Scopus, Web of Science and ScienceDirect.

The second phase of the search will be conducted on the Google® Scholar search engine and with the support of the Publish or perish [19] software, using the terms indexes and synonyms (keywords) identified in the studies in the initial phase of the research. which retrieved all citations and results in a static moment, for the purpose of exporting to analysis software. Finally, the third and final phase involved searching the references of studies selected in the previous phases.

### Search strategy

For the search, the following descriptors were used: “Mobile applications”, “Mobile applications”, “Aplicaciones Móviles” and “Enfermagem”, “Nursing” and “Enfermería”, obtained from the Medical Subject Heading (MeSH) and the Descriptors page of Health Sciences (DeCS). These descriptors resulted in crossings using the Boolean operators AND and OR, according to the following syntax presented below: 1# AND 2# (Appendix S1 File).

To ensure consistency in results, the studies will be collected in a single day, using the Academic Federated Community (CAFe) of the Periodicals Portal of the Coordination for the Improvement of Higher Education Personnel (CAPES), which gives access to some articles, periodicals and data sources for free.

### Selection of sources of evidence

The results obtained from the data sources will be exported to the research support tool Rayyan [20]. On this platform, two researchers will independently and blindly conduct the initial selection of studies, based on the analysis of their titles and abstracts. After ensuring agreement between the two researchers regarding the selected studies, they will be examined in full and included in the sample.

In situations where disagreements arise, a third guest researcher will be consulted to resolve the issue. The entire process follow the PRISMA-ScR flowchart, and will be properly documented to allow detailed monitoring of the selection process.

## Inclusion of additional sources

After collecting data in the first and second phase, researchers will analyze the references of the studies initially collected in order to add new studies to the sample, complying with the recommended saturation.

### Second pass: full-text screening

The sample studies will be divided equally among researchers so that they can read them in full and extract the study variables.

### Data extraction

Data from the studies will be collected using an extraction tool adapted from the model proposed by JBI for scoping reviews [16].

The data will be stored in an electronic spreadsheet linked to Microsoft Excel, and organized according to the variables described in the protocol, including identification number, data source, title, type of study, country of publication, language(s), year of publication, authors, level of evidence and application. Furthermore, according to the proposed objective, the studies - and consequently, the mobile applications in nursing graduation - will be analyzed according to SWOT, which consists of a strategic tool composed of four elements: strengths and weaknesses, which concern internal and controllable aspects, and opportunities and threats, which are related to external and non-controllable factors [18].

Strengths represent positive, intangible internal attributes that are under the control of the organization. On the other hand, weaknesses are negative attributes that can hinder the ability to achieve established goals [18,21].

Opportunities refer to attractive external factors that can be leveraged for the benefit of the organization. Finally, threats are external and uncontrollable attributes that represent potential risks to the organization’s purpose [18,21].

A pilot test of data extraction will be conducted on a random sample of 25 studies to ensure that the extracted variables meet the purpose of the review. The entire research team involved in this review will examine the instrument according to the eligibility criteria and the variables selected for the study. Discrepancies will be discussed and modifications to the instrument variables will be made.

### Analysis of evidence

Finally, following all processes and eligibility criteria, the studies will make up the sample. These will undergo a level of evidence analysis, following guidelines the JBI approach classification will be adopted: levels of evidence, where studies are evaluated as follows: level 1 – experimental studies; level 2 – quasi-experimental studies; level 3 – analytical observational studies; level 4 – descriptive observational studies; level 5 – expert opinion and bench research [22]. This type of analysis will be carried out to fairly discuss the results of the studies according to the level of evidence presented. Furthermore, this makes it easier for readers to understand the level of evidence to base practice on.Thematic analysis of the results will also be carried out to list them according to the SWOT tool. The concepts used to define each element of the SWOT acronym will be the same as those described in the data extraction section above.

### Ethical Aspects

The research did not involve human beings, therefore, it was not necessary to refer it to the Research Ethics Committee.

## Results

The results will cover the characterization of the sample studies, followed by a SWOT chart containing the main strengths, weaknesses, opportunities and threats of mobile applications in undergraduate nursing.

To this end, the results will be presented graphically through tables and charts and in a descriptive manner, in order to answer the research questions, and the selected studies will be arranged by figure according to the Preferred Reporting Items for Systematic Reviews and Meta-Analyses, added to a narrative process of the findings.

## Discussion

The use of mobile applications has been gaining prominence at an increasing pace, particularly in higher education institutions. This highlights possibilities for improving more significant results, especially in terms of mobility, enabling students and teachers to share information at the same time and in any scenario [23].

In the educational sphere, technologies in general have the potential to promote a new learning environment, in a way that can promote student autonomy, collaboration and vivid and active participation. At the same time, it contributes to reorienting the role of the teacher, who is responsible for mediating the education process using technological resources in the development of educational materials [24].

The review will allow for the provision of comprehensive information about the use of technologies, particularly applications in use linked to nursing degrees. Along with this, you will be able to map evidence regarding the main strengths, weaknesses, opportunities and threats associated with mobile applications used in teaching. It can also assist in investigating the knowledge gap in the literature and where original research or more robust reviews are needed.

The results will also present the characterization of the studies that made up the sample and through a table in figure format containing the strengths, weaknesses, opportunities and threats of mobile applications at the heart of undergraduate nursing. The use of the SWOT strategy can mean a new and differentiated methodology for analyzing concepts and study variables, allowing the synthesis and visualization of parameters of interest.

Among the limitations involving the review, it is worth highlighting the impossibility for researchers to access some of the studies because they are paid for, as well as the impossibility of evaluating the quality of the studies that made up the sample individually. From this perspective, it is recommended that systematic reviews and meta-analyses be carried out with the aim of comparing methodologies and interventions used in the study context.

## Conclusion

By identifying the strengths, weaknesses, opportunities and threats of the object of study, researchers gain self-knowledge and can strategically plan the development and implementation of mobile applications in undergraduate nursing. Furthermore, SWOT analysis assists in making informed decisions, communicating effectively with funders and collaborators, and planning for publishing and disseminating relevant and impactful results. This contributes to improving the quality and positive impact of scientific research.

## Supporting information

S1 Checklist PRISMA-P 2015 checklist. (PDF)

S1 File Appendix for Search strategies for each database. (DOCX)
