# Supplementary material for: Strengths, weaknesses, opportunities and threats of mobile applications in undergraduate nursing: a scoping review protocol
Source: PLoS One. 2025 Apr 3;20(4):e0314757. doi: 10.1371/journal.pone.0314757 (PMC11967970; doi:10.1371/journal.pone.0314757)
Supplement: S1 File — (DOCX) [file pone.0314757.s002.docx]

**Table 1. Description and characterization of the PCC mnemonic used in the research.**

| **Population/Problem:** Undergraduate nursing students and teachers | Individuals enrolled in higher-level courses, which are offered by educational institutions, where students receive theoretical and practical training to become nursing professionals and teachers are responsible for the content taught and the development of professional practice. |
| --- | --- |
| **Concept:** Strengths, weaknesses, opportunities and threats | Strengths: represent positive and intangible internal attributes, which are under the control of the organization.  Weaknesses: these are negative attributes that can hinder the ability to achieve established objectives.  Opportunities: refer to attractive external factors that can be used to benefit the organization.  Threats: are external and uncontrollable attributes that represent potential risks to the organization's purpose.(18) |
| **Context:** Mobile applications | Software programs designed specifically to run on mobile devices, such as smartphones and tablets, that provide services and functionality to users, making it easier to perform tasks, access information and communicate.(4) |
